# Influence of age and seasonality on boar seminal plasma steroids quantification: A preliminary study

**DOI:** 10.14202/vetworld.2023.2150-2157

**Published:** 2023-10-21

**Authors:** Camilla Aniballi, Alberto Elmi, Nadia Govoni, Tiziana Bulla, Elena Canelli, Antonio Casalini, Maria Laura Bacci, Domenico Ventrella

**Affiliations:** 1 Department of Veterinary Medical Sciences, University of Bologna, via Tolara di Sopra 50, 40064, Ozzano dell’Emilia (BO), Italy; 2 Department of Statistical Sciences, University of Bologna, Via Belle Arti, 41, 40126, Bologna, Italy; 3 Swine Practitioner, PBA s.r.l., Via Gerole, 1, 26861, Fombio, LO, Italy

**Keywords:** boar, ejaculate, reproduction, seasonal parameters, steroids, swine

## Abstract

**Background and Aim:**

Seasonal changes, especially temperature and photoperiod, are well-known determining factors of swine reproductive capacity, but the underlying mechanisms remain unknown. This study aimed to investigate the effect of age and seasonal variations on boar seminal plasma steroids (dehydroepiandrosterone [DHEA], cortisol [CORT], and testosterone [TEST]) over 1 year.

**Materials and Methods:**

Four commercial hybrid adult boars (Large White × Duroc), aged between 12 and 44 months, were repeatedly evaluated at the Department of Veterinary Medical Sciences of the University of Bologna. Daily temperature and light hours relating to the collection date were considered for each observation within the four astronomical seasons: Winter, spring, summer, and autumn. Hormones were quantified using radioimmunoassay. The association between seasonal factors and hormone concentrations was evaluated using linear regression models. Univariate models were estimated for each hormone to assess the influence of the independent variables; two multivariate models were assessed to evaluate the effect of temperature and daylight hours, including boar and season factors.

**Results:**

Age significantly affected all analyzed hormones (CORT p < 0.0001; DHEA p < 0.0001; and TEST p < 0.0001). The highest average levels were found for each hormone during summertime, suggesting a positive correlation between steroid concentrations with temperature and light hours.

**Conclusion:**

The results of this study support the hypothesis that the increase in external temperature and light hours is somehow associated with higher levels of steroid concentrations in the seminal plasma of in-housed boars. These findings may help further investigate seasonal fluctuations in reproductive outcomes, which are well-known for porcine species.

## Introduction

During ejaculation and deposition in the female genital tract, spermatozoa are carried by the seminal plasma, a mixture of secretions produced by the accessory sex glands [1]. Mainly consisting of proteins [2], the seminal plasma also contains inorganic ions, salts, sugars, citric acid, prostaglandins, and electrolytes [3]; however, its multiple functions remain unclear. Seminal plasma proteins are the most studied components for different functions and the presence of prostasomes or other molecules [4]. Despite their lipophilic nature and many possible roles in reproduction, steroid hormones in seminal plasma, including sexual steroids, have received less attention. Seminal plasma generally aids sperm survival and fertility in several species, providing energy for metabolism and motility and buffering against pH changes. It also regulates and controls the capacitation phenomenon and aids transport through the female tract, which is essential for fertilization [1, 5]. However, its role in swine reproduction is controversial because it is suspected to increase oxidative stress and negatively affect *in vitro* sperm survivability in a boar-dependent manner [6, 7]. At present, most sows in intensive farming conditions undergo artificial insemination (AI). Knowledge of the factors influencing semen quantity and quality is pivotal for improving AI in zootechnical production. Boars produce a broad spectrum of steroids, most of which are of testicular origin [8]. These hormones include testosterone (TEST), dehydroepiandrosterone (DHEA), and cortisol (CORT). Among them, DHEA is an endogenous steroid hormone precursor of androgens and estrogen in males and females, respectively; it is synthesized from cholesterol in the cortical layer of the adrenal glands, gonads, and brain [9]. Evidence suggests that DHEA also has neuroprotective and antiglucorticoid effects and influences animal behavior by decreasing aggressiveness [10]. Cortisolis a glucocorticoid synthesized by the cortex area of the adrenal gland, which is the last unit of the hypothalamic–pituitary–adrenal axis. Cortisol plays a pivotal role in responding to different physiological or stressful conditions, increasing the catabolism of tissues rich in protein and fat to produce glucose; therefore, it is often called the “stress hormone” [11]. Testosterone is an androgen obtained from cholesterol; in males, it is mainly produced by the testes, predominantly released by Leydig cells, with small amounts secreted by the adrenal glands [12]. Testosterone circulating concentrations increase after puberty, influencing the expression of sexual behaviors and the development of secondary sexual organs [13].

Seasonal changes, especially temperature and photoperiod, are well-known determining factors of swine reproductive capacity. Spermatozoa quantity, and thus boars’ fertility, is significantly influenced by season, with higher values in winter and lower in summer [14–16]. The volume collected, percentage of live sperm, and motility score tended to be highest in autumn and winter, coincident with decreasing daylight hours and temperature [14]. Autumnal reduction in daylight generally stimulates boars’ reproductive functions [15, 16]. The influence of seasonality has also been discussed with reference to another factor closely related to reproduction, such as steroid hormones, which can regulate animals’ reproductive capabilities [12, 17, 18]. Seasonal influences on steroids are common for wild mammals when addressing ruminants, generally considered to be the most linked to environment and photoperiod, and wild boars [19–22]. The breeding period of the wild boar coincides with the late autumn and winter months, with a secondary mating season in the spring [22]. However, the domestic pig under farming conditions reproduces throughout the year [16]. In intensive farm settings, because the reproductive life span of a boar breeder is very short, there are no accurate studies on the fertility progress of animals of a greater age [23]. Despite the highly standardized environmental conditions in which boars are usually housed within breeding facilities, seasonal changes are relevant in determining fertility rates [24].

To better characterize the influence of animal- and environmental-related parameters on porcine reproductive capabilities, this study aimed to evaluate the association between age, season (in terms of outdoor environmental daylight hours and temperature), and steroid concentration in boar seminal plasma by evaluating the varying levels of three different hormones: DHEA, CORT, and TEST.

## Materials and Methods

### Ethical approval

Semen collection for reproductive/experimental purposes does not qualify as a procedure, according to National legislation (D.lgs 26/2014), therefore no ethical approval was needed for this study.

### Study period and location

This study was conducted from March 2016 to March 2017 at the Department of Veterinary Medical Sciences of the University of Bologna (Ozzano dell’Emilia, BO, Italy).

### Study design

This prospective analysis was based on opportunistic observations of seminal plasma samples collected from four commercial hybrid adult boars (Large White × Duroc). Semen collections covered different ages of the boars: Boar A was sampled between 14 and 22 months of age, Boar B between 35 and 44, Boar C between 33 and 35, and Boar D between 12 and 20. The animals were housed in single-pen indoor and regularly monitored for good health, according to the National Law for Animal Welfare Good Practices (D.lgs n. 122/2011). They were fed a commercial diet specifically developed for boars (3000 kcal/kg of feed), split into two daily administrations (08:00 am and 04:00 pm). According to individual weight, animals received between 3 and 3.5 kg/die of feed. Environmental conditions were artificially set to meet the boars’ requirements: The light/dark cycle was set at 12/12 h with a minimum Lux value of 40 during light hours, and the temperature was set at 21°C ± 1°C. Data recorded for each sampling included in this study were boar ID and age (months), season, external temperature (°C), daylight hours (h) at the time of collection, and the DHEA, CORT, and TEST concentrations. External temperature and daylight hours were used as season-related variables to allow for more comprehensive and accurate statistical modeling.

### Seminal plasma and seasonal factors

Semen was manually collected to high hygienic standards using the hand-gloved technique in a preheated (37°C) thermos. All sampling procedures were conducted between 9:00 and 10:00 am. On collection, the ejaculate was processed at the analysis laboratory, aliquoted, and centrifuged (1200× *g* for 10 min, RT) to isolate the boar seminal plasma from spermatozoa. Samples were stored at −20°C, until analysis (maximum storage time: 40 months). Regardless of sample inclusion in this study, boars were sampled twice weekly.

The mean temperature and light hours relating to the date and location of the collection were recorded for each observation from the Arpae Emilia Romagna website [25] and used as continuous variables to describe the given season.

### Steroids (CORT, DHEA, and TEST) extraction and quantification

Cortisol, DHEA, and TEST were extracted by mixing 1 mL seminal plasma with 10 mL diethyl ether for 1 h on a rotary shaker, as described by Gaiani *et al*. [26], with partial modifications in terms of volume of samples, still maintaining the correct ratio. The tubes were centrifuged at 3000× *g* for 15 min, the supernatants were evaporated to 37°C under an air-stream suction hood, and the dry residues were stored at −20°C until analysis (maximum storage time: 2 weeks). Three additional samples spiked with a small amount (≈1000 CPM, counts/min) of the tritiated hormones were used to determine extraction recovery. The samples were vortexed and equilibrated for 2 h before solvent extraction. The mean percentage of recovery was 89.61% ± 1.48%.

The dry extracts were reconstituted in assay buffer (phosphate-buffered saline, 0.1% bovine serum albumin, pH = 7.4) for the measurement of CORT (100 μL seminal plasma equivalent), TEST, and DHEA (200 μL seminal plasma equivalent) using radioimmunoassay; tritiated CORT (30 pg/tube; 94.6 Ci/mmol; PerkinElmer, USA), tritiated TEST (30 pg/tube; 83.4 Ci/mmol; PerkinElmer), or tritiated DHEA (30 pg/tube; 76.1 Ci/mmol; PerkinElmer) were added, followed by rabbit anti-CORT serum (0.1 mL, 1:20000; produced in-house), rabbit anti-TEST serum (0.1 mL, 1:50,000; produced in-house), or rabbit anti-DHEA serum (0.1 mL, 1:10,000; produced in-house), respectively. After incubation and separation of antibody-bound and -unbound steroid using charcoal-dextran solution (charcoal 0.25%, dextran 0.02% in phosphate buffer), tubes were centrifuged (15 min, 3000 g), the supernatant was decanted, and radioactivity was immediately measured using a β-scintillation counter (Packard C1600, PerkinElmer).

The sensitivity of the CORT assay was 4.13 pg/tube, and the intra- and inter-assay coefficients of variation were 4.9% and 9.2%, respectively. The sensitivity of the TEST assay was 3.08 pg/tube, and the intra- and inter-assay coefficients of variation were 5.7% and 10.5%, respectively. The sensitivity of the DHEA assay was 3.2 pg/tube, and the intra- and inter-assay coefficients of variation were 4.9% and 8.7%, respectively. Cross-reactions of various steroids with antiserum raised against CORT were: CORT 100%, cortisone 5.3%, 11α-deoxycortisol 5.0%, corticosterone 9.5%, 20α-dihydrocortisone 0.4%, prednisolone 4.60%, progesterone < 0.001%, and TEST < 0.001%. Cross-reactions of different steroids with antiserum raised against TEST were: TEST 100%, dihydrotestosterone 25.44%, androstenedione 0.6%, DHEA 0.2%, and progesterone and CORT < 0.0001%. Cross-reactions of different steroids with antiserum raised against DHEA were: DHEA 100%, DHEA sulfate 39%, androstenedione 10%, TEST 0.25%, and progesterone and CORT < 0.001%. To determine the parallelism between hormone standards and endogenous hormones in boar seminal plasma, a pooled sample containing high CORT, DHEA, and TEST concentrations was serially diluted (1:1–1:8) using assay buffer. A regression analysis was used to determine the parallelism between the two hormone levels in the same assay, and a high degree of parallelism was confirmed (r^2^ = 0.98). The assay results are expressed as ng/mL.

### Statistical analysis

Statistical analyses were performed using the software R version 3.6.3 (The R Foundation for Statistical Computing, Vienna, Austria). Graphic representations were obtained using GraphPad Prism v.9 (GraphPad Software Inc., San Diego, CA, USA). To assess the effect of age and season on hormone concentrations, non-parametric Kruskal–Wallis tests followed by Dunn’s multiple comparison tests were used (p < 0.05). The association between seasonal factors and the concentration of each steroid hormone was assessed using linear regression models; season, temperature, and light hours were recorded as independent variables. Hormone variables were transformed because of their non-normal distribution (Shapiro–Wilk normality test, p < 0.05); logarithmic transformations were applied for DHEA and TEST, whereas a square root (SQRT) transformation was used for CORT. The correlation between the variables was evaluated using Spearman’s rank correlation coefficient. Univariate models were estimated for each hormone concentration (TEST, CORT, and DHEA) to evaluate the influence of the independent variables; two multivariate models were then used to separately evaluate the effect of temperature (Model 1) and daylight hours (Model 2), including boar and season factors. The temperature and daylight hours were included separately to limit the presence of a strong correlation between two or more independent variables in the same model (multicollinearity phenomenon).

## Results

### Observations and hormonal quantification

This study included 76 semen samples, called observations, collected from 4 boars: 23 observations for the first boar (Boar A), 19 for the second (Boar B), 5 for the third (Boar C), and 29 for the fourth (Boar D). The different contributions from the four boars, in terms of analyzed samples, are because of the study’s opportunistic and retrospective nature. Samples were obtained during a longer time span covering different ages of boars, as previously reported.

Among the 76 observations, 10 were in winter, 18 in spring, 21 in summer, and 27 in autumn. The analytical methodology allowed for the reliable quantification of the three chosen hormones in the collected samples. Table-1 shows the mean hormone levels along with standard deviations grouped by boar, age, and season.

**Table-1:** Descriptive statistics (mean ± SD) of the three analyzed hormones grouped by boar, age, and season.

Variable	n	CORT ng/mL	DHEA ng/mL	TEST ng/mL
Boar				
*BoarA*	23	0.83 ± 0.56	0.28 ± 0.17	0.46 ± 0.26
*BoarB*	19	0.26 ± 0.13	0.15 ± 0.10	0.20 ± 0.13
*BoarC*	5	1.26 ± 0.97	0.27 ± 0.16	0.31 ± 0.21
*BoarD*	29	1.00 ± 0.42	0.37 ± 0.20	0.45 ± 0.22
Age (years)				
1 year	4	1.46 ± 0.30	0.67 ± 0.31	0.68 ± 0.35
2 years	48	0.88 ± 0.48	0.30 ± 0.15	0.44 ± 0.22
3 years	9	0.78 ± 0.89	0.20 ± 0.14	0.24 ± 0.18
4 years	15	0.29 ± 0.13	0.16 ± 0.11	0.21 ± 0.13
Season				
Winter	10	0.91 ± 0.71	0.18 ± 0.09	0.31 ± 0.15
Spring	18	0.67 ± 0.68	0.31 ± 0.28	0.34 ± 0.30
Summer	21	0.92 ± 0.58	0.33 ± 0.16	0.48 ± 0.27
Fall	27	0.71 ± 0.38	0.27 ± 0.14	0.36 ± 0.19

CORT=Cortisol, DHEA=Dehydroepiandrosterone, TEST=Testosterone, SD=Standard deviation

### Age and season

**Figure-1:**
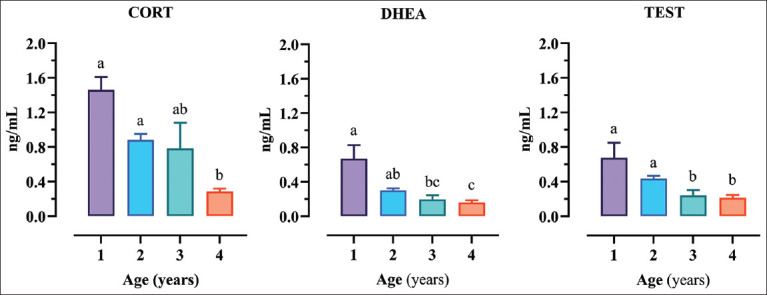
Graphical representation of the influence of age on hormone levels. Histograms and error bars represent means and standard error of the mean. Different superscript letters indicate the results of the *post hoc* Dunn’s tests (p < 0.05).

Regarding age, the Kruskal–Wallis analysis showed how it significantly affected all analyzed hormones (CORT p < 0.0001; DHEA p < 0.0001; and TEST p < 0.0001). The results of the *post hoc* Dunn’s multiple comparison tests are represented in Figure-1 using different superscript letters. In general, relevant differences started appearing in the 3^rd^ year of life.

**Figure-2:**
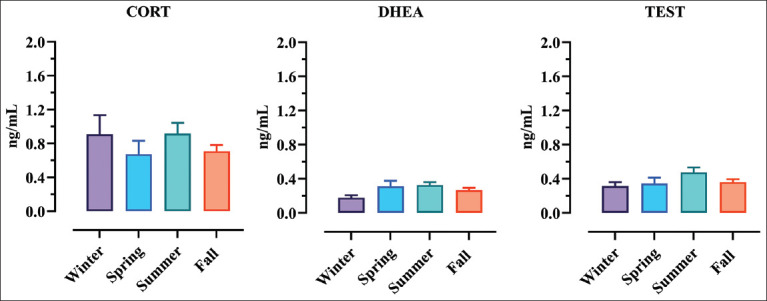
Graphical representation of the influence of the season on hormone levels. Histograms and error bars represent means and standard error of the mean.

Regarding season, the Kruskal–Wallis analysis showed no statistically relevant difference for the three tested hormones (CORT p < 0.2520; DHEA p < 0.1200; and TEST p < 0.1650), as shown in Figure-2.

### Cortisol

Table-2 shows the regression coefficients and p-values derived from the linear regression models estimated for the variable SQRT of CORT; Model 2 shows a positively significant association between CORT levels and daylight hours (coefficient = 0.07, p = 0.004).

**Table-2:** Regression coefficients and p-values of the statistical models for CORT.

Variable	Univariate analysis		Model 1 Temperature		Model 2 Daylight hours	
		
	Coefficient	p-value	Coefficient	p-value	Coefficient	p-value
Boar						
*BoarA*	ref	-	ref	-	ref	-
*BoarB*	−0.36	<0.001	−0.43	<0.001	−0.47	<0.001
*BoarC*	0.16	0.208	0.14	0.284	0.13	0.317
*BoarD*	0.12	0.105	0.11	0.112	0.09	0.170
Season						
*Winter*	ref	-	ref	-	ref	-
*Spring*	−0.18	0.157	−0.21	0.112	−0.34	0.014
*Summer*	0.01	0.915	−0.17	0.330	−0.20	0.132
*Autumn*	−0.09	0.447	−0.24	0.026	−0.13	0.147
Temperature	0.003	0.461	0.01	0.09	-	-
Daylight hours	0.008	0.620	-	-	0.07	0.004

CORT=Cortisol

Compared with the summer season, the CORT measurements made in spring/autumn/winter tend to be averagely lower but not statistically significant (univariate analysis); temperature and daylight hours adjusted Models, 1 and 2, respectively, show significance only for the latter.

### Dehydroepiandrosterone

Table-3 shows the regression coefficients and p-values derived from the linear regression models estimated for the log variable DHEA; the multiple regression models suggest a significantly positive association of DHEA levels with temperature (Model 1, coefficient = 0.03, p = 0.028) and daylight hours (Model 2, coefficient = 0.12, p = 0.02).

**Table-3:** Regression coefficients and p-values of the statistical models for DHEA.

Variable	Univariate analysis		Model 1 Temperature		Model 2 Daylight hours	
		
	Coefficient	p-value	Coefficient	p-value	Coefficient	p-value
Boar						
*BoarA*	ref	-	ref	-	ref	-
*BoarB*	−0.62	<0.001	−0.88	<0.001	−0.94	<0.001
*BoarC*	−0.01	0.976	−0.03	0.923	−0.07	0.803
*BoarD*	0.30	0.054	0.21	0.156	0.17	0.231
Season						
*Winter*	ref	-	ref	-	ref	-
*Spring*	0.28	0.274	0.12	0.655	0.05	0.868
*Summer*	0.56	0.025	−0.01	0.985	0.16	0.565
*Autumn*	0.35	0.145	−0.05	0.818	0.19	0.334
Temperature	0.02	0.019	0.03	0.028	-	-
Daylight hours	0.04	0.156	-	-	0.12	0.02

DHEA=Dehydroepiandrosterone

The summer higher DHEA concentrations that emerged in the Univariate Analysis (coefficient = 0.56, p = 0.025) were not confirmed when the effect was adjusted in Models 1 and 2 for temperature and light hours, respectively.

### Testosterone

Table-4 shows the regression coefficients and p-values derived from the linear regression models estimated for the log variable TEST, suggesting a significantly positive association of TEST levels with daylight hours (Model 2, coefficient = 0.12, p = 0.021), while the association with temperature is weaker (Model 1, coefficient = 0.03, p = 0.057).

**Table 4:** Regression coefficients and p-values of the statistical models for TEST.

Variable	Univariate analysis		Model 1 Temperature		Model 2 Daylight hours	
		
	Coefficient	p-value	Coefficient	p-value	Coefficient	p-value
Boar						
*BoarA*	ref	-	ref	-	ref	-
*BoarB*	−0.79	<0.001	−1.01	<0.001	−1.06	<0.001
*BoarC*	−0.36	0.176	−0.40	0.147	−0.43	0.110
*BoarD*	0.05	0.728	−0.01	0.965	−0.04	0.789
Season						
*Winter*	ref	-	ref	-	ref	-
*Spring*	−0.09	0.716	−0.13	0.612	−0.25	0.360
*Summer*	−0.37	0.122	−0.12	0.729	−0.03	0.902
*Autumn*	0.13	0.579	−0.28	0.191	−0.07	0.728
Temperature	0.02	0.069	0.03	0.057	-	-
Daylight hours	0.02	0.428	-	-	0.12	0.021

TEST=Testosterone

## Discussion

Analyzing the factors influencing seminal plasma characteristics in farm animals can be particularly relevant, especially for the zootechnical industry. Other than individual-specific quality, possible associations between seminal plasma traits and environmental or seasonal factors have already been investigated. Indeed, despite modern piggeries being capable of artificially managing daylight hours and temperature, the same seasonal factor variation still impacts swine reproduction [24]. Of the different methodologies currently used for quantifying steroids in biological matrices, radioimmunoassay (RIA) was chosen for various reasons, including the years of experience of the research team. RIA protocols also allow for accurate and reliable results with high sensitivity compared with mass spectrometry technologies [27].

The observational nature of this study constitutes its main limitation. The analyses were performed on seminal plasma samples stored for other experimental purposes, and sampling schemes were not designed. All information regarding the frequency of samplings and animals’ contribution to the study are clearly stated for transparency and correct reporting of scientific studies. Therefore, it is appropriate to remember the difficulties often encountered in smaller farming systems, as opposed to genetic centers, where the number of boars is generally small, and it is often impossible to perform continuous sampling. Therefore, the animals were sampled at different times, and their reproductive physiology was affected by seasonal features. These features require appropriate statistical approaches such as multiple regression models, that simultaneously consider the different factor effects. The previous study by Macchi *et al*. [22] demonstrated that the season could influence the reproductive capacity and concentration of boar’s fecal and plasmatic steroid hormones, with higher values in winter. Our study did not significantly highlight this association after measuring the concentrations of three steroid hormones, DHEA, CORT, and TEST, in the seminal plasma of four bears and evaluating their seasonal variations. Using linear regression models, the statistical estimations suggested a positive relationship between each steroid concentration with environmental temperature and light hours. These relationships emerged clearly for DHEA, CORT, and TEST, with weaker evidence for temperature. If the association between boar seminal plasma steroids and temperature seems slightly discordant in the literature results, the positive relationship with light hours results from greater consistency. Claus *et al*. [8] showed a significant increase in boars’ hormone concentrations when exposed to a more extended period of light.

The previous study by Collomp *et al*. [28] have shown how sex and age affect plasma DHEA concentrations. Moreover, DHEA and CORT release in healthy subjects are apparently synchronous. In humans, DHEA displays diurnal patterns of secretion, resembling those of TEST and CORT in some circumstances; nevertheless, the various biological actions of DHEA in different mammal species remains unclear [29]. One notable function of this steroid is its role as a prohormone used by many nonendocrine peripheral tissues to synthesize androgens and estrogens. In addition, the most intriguing aspect of DHEA is its anti-inflammatory action. It is involved in the downregulation of the complement cascade and inflammatory cytokines and the upregulation of anti-inflammatory interleukin-2 synthesis; it opposes glucocorticoid effects [30]. It was hypothesized that DHEA and CORT in seminal plasma could be immunomodulatory molecules toward the immune cells patrolling the female reproductive tract [31].

The previous studies by Claus *et al*. [8] and Macchi *et al*. [22] have shown that seasonality significantly influences the quantitative and qualitative features of swine circulating blood steroids, with higher values in colder months, between November and January, and lower values between April and June. Because of their lyophilic nature, locally synthesized steroids are not confined to the originating tissue, and their concentration in specific organs, such as the testis, can be influenced by circulating levels. Human plasma contains two steroid-binding proteins: Corticosteroid-binding globulin (CBG) and sex hormone-binding globulin (SHBG) [32]. Corticosteroid-binding globulin is the primary CORT-binding protein and represents an eclectic component in the mechanisms of hypothalamic–pituitary–adrenal axis–related homeostasis [33]. Approximately 90% of secreted CORT is bound to CBG, whereas the remaining 5% of free CORT is considered the biologically active fraction. Corticosteroid-binding globulin acts as a modulator allowing the release of CORT to different tissues [34]. Corticosteroid-binding globulin is sensitive to temperature fluctuations, releasing CORT in response to fever and external sources of heat [35]. Seasonality temperature changes could affect organ/tissue-specific delivery of circulating CORT, causing a slightly increased CORT seminal plasma fraction.

Testosterone circulates through vessels prevalently bound to a specific globulin (SHBG) that is biologically active, differently from bounded CORT. Some studies have hypothesized that the TEST-bounded fraction could be available to specific tissues, such as the prostate gland and the testis, through active transport [36, 37]. Therefore, the TEST concentration in seminal plasma represents the combination of locally synthesized and blood-circulating TEST, free, and bounded fractions. Reduced daylight during fall generally causes physiological changes that stimulate reproductive functions in boars. In summer, steroid concentration reduction may be related to lower fertility caused by heat stress. Therefore, it could be assumed that seasonality influences blood steroids differently than seminal plasma, as seen in this study. The seasonal effects can be observed even if animals are housed in enclosed standardized environments. Indeed, while the warmer seasons highlighted a slight increase in seminal plasma steroids, the previous research showed the opposite occurrence concerning other matrices [8, 18, 22].

Limitations regarding the sample size, the unbalanced study design, and the impossibility of separating the effect of temperature from that of daylight hours should be further investigated. The general lesson learned from humans is that acute and chronic stressful situations or diseases can affect plasma steroid concentrations. Glucocorticoids regulate their secretion through a feedback inhibition mechanism. However, the stress response is not merely related to HPA axis activation; other factors help animals adapt to stressors [11]. Stressors could affect DHEA secretion through indirect endocrine mechanisms, and the physiological reaction could be better described through a simultaneous expression of both steroids, such as the CORT/DHEA ratio, which may be an indicator of the animal response. Regarding TEST, although a reduction in the reproductive performances of boars during the summer season is yet to be highlighted [22], its elevated levels in seminal plasma could be due to the prohormone effect of DHEA. Therefore, it could be assumed that the increase in DHEA levels might be more functional to its local anti-inflammatory action than to its reproductive role, justifying why previous studies reported low plasma concentrations of both steroids, DHEA and TEST during summer. Therefore, the higher TEST levels measured in the summer could be a nonfunctional consequence of this DHEA effect. Concerning the age of the boars, the highest levels of seminal plasma steroids (CORT, DHEA, and TEST) were observed in their 1^st^ year of life, reducing as the subjects got older. These data conform with previous studies’ results on blood sexual steroids, because when sexual maturity occurs, a peak production of sexual steroid hormones is achieved [38, 39].

For future studies, extending the samplings throughout the seasonal transition would be highly interesting to compare the CORT, DHEA, and TEST levels between blood and seminal plasma. In addition, it would be pivotal to analyze ejaculates for morphofunctional parameters indicative of fertility to understand the effects of steroid levels on viability, motility, and acrosomal status. In this case, multiple blood samplings to evaluate plasmatic steroids were not performed because of the absence of an experimental protocol approved by the National Ethics Committee, and seminal quality evaluations were excluded because they were not performed on all samples.

## Conclusion

Despite some interpretative complications and the nonlinearity of some associations, the results of this study verify the first descriptive idea, according to which the increase in external temperature and light hours is somehow associated with higher levels of steroid concentrations in the seminal plasma of in-housed boars. To better understand the effects of said higher hormone levels in seminal plasma, further studies are required to highlight potential correlations with male fertility parameters, such as sperm viability, motility, and acrosomal status. In addition, searching for parallelisms with blood levels would be pivotal to gain a more comprehensive picture of the hormonal profile. These future steps may be critical to better understand and unravel the seasonal hypofertility, typical of the warmed months, well known for porcine species.

## Authors’ Contributions

MLB, DV, and AE: Conceptualization. CA, NG, and AC: Methodology. TB, AE, and EC: Formal analysis. CA: Drafted the manuscript. AE and DV: Revised the manuscript. MLB and EC: Supervised the study. All authors have read, reviewed, and approved the final manuscript.
